# The significance of the library's physical space: how COVID-19 impacted a consumer health service

**DOI:** 10.5195/jmla.2023.1420

**Published:** 2023-04-21

**Authors:** Kelsey L. Grabeel, Cameron Watson, Alexandria Q. Wilson

**Affiliations:** 1 kgrabeel@utmck.edu, Associate Professor, Health Information Center, Preston Medical Library, University of Tennessee Medical Center / University of Tennessee Graduate School of Medicine, Knoxville, TN.; 2 cwatson1@utmck.edu, Library Associate II, Health Information Center, Preston Medical Library, University of Tennessee Medical Center / University of Tennessee Graduate School of Medicine, Knoxville, TN.; 3 aqwilson@utmck.edu, Assistant Professor, Health Information Center, Preston Medical Library, University of Tennessee Medical Center / University of Tennessee Graduate School of Medicine, Knoxville, TN.

**Keywords:** Consumer health information, hospital library, COVID-19, physical space

## Abstract

**Background:**

During the beginning of the COVID-19 pandemic, many consumer health libraries were forced to close their doors to patrons. At the Health Information Center in Knoxville, Tennessee, the physical space closed, while health information services continued to be provided via phone and email. To examine the impact of lack of access to a physical library for consumer health information, researchers analyzed the number of health information requests pre-COVID-19 pandemic compared to during the initial phase of the pandemic.

**Case Presentation:**

Data from an internal database was collected and analyzed. Researchers divided the data into three time periods: March 2018 to February 2019 (Phase 1), March 2019 to February 2020 (Phase 2), and March 2020 to February 2021 (Phase 3). Data was de-identified and duplicate entries were removed. The type of interaction and request topics were reviewed in each phase.

**Conclusion:**

In Phase 1, there were 535 walk-ins to request health information and 555 walk-ins in Phase 2. In Phase 3, there were 40 walk-ins. The number of requests through phone and email varied but remained steady. There was a 61.56% decrease in requests between Phase 1 and Phase 3 while there was a 66.27% decrease between Phase 2 and Phase 3 due to the lack of walk-in requests. The number of phone and email requests did not increase despite the closure of the physical library space to the public. Access to the physical space plays a significant role in providing health information requests to patients and family members.

## BACKGROUND

With the changing roles of libraries and the persistence of the digital divide, or “the gap between people who can easily use and access technology, and those who cannot” [1], the physical space of the library is more important than ever as it provides people in the gap the ability to access information, whether it be with new technology or how to find health information [2, 3]. This is especially true for hospital libraries, which provide valuable services not only to physicians and nurses but also to consumers. It can be difficult for consumers to find quality health information and the accuracy of these resources may vary [4, 5]. Hospital libraries can play a crucial role in providing evidence-based medicine to both the public and healthcare providers [6]. Furthermore, the library can help consumers find quality health information they are looking for through a consumer health information service. By having a physical hospital library, consumers have an opportunity to walk into the space, speak with a librarian, learn more about how to access resources, and potentially ask for health information [7].

“Libraries as space” studies have examined academic libraries and discussed public libraries [8], but few have focused on hospital library space for patients, caregivers, and other consumers [9–12]. Research has found that some hospital libraries had patients/caregivers as their most frequent users [9] while others were able to add a consumer health library to their already established physical space, which led to increased foot traffic for some [10]. Health sciences libraries, including hospital libraries, have been closing due to budget cuts or mergers [11, 12]. One study found that in 1989 about 44% of hospitals had an onsite library; however, that number dropped to 33.5% and 29.1% in 2005-2006 [12]. Furthermore, the Joint Commission on Accreditation of Healthcare Organizations (JCAHO) does not state that hospitals need to have a library or librarian [13], which highlights that librarians are not seen as essential for patients and family members at hospitals. Hospital management may not see the benefits of having a physical library, which could lead to further closures.

The fear of permanent closure may have increased in 2020 due to the COVID-19 pandemic when many libraries were forced to close their physical spaces. As a result, libraries increased their remote services, while also helping patients navigate through all the COVID-19 information [14]. Although these closures were essential to help “flatten the curve” of COVID-19 [15], they eliminated the potential walk-ins of patients and their family members inquiring about health information and shifted the focus to providing health information only virtually to the community. Virtual libraries became available for the community to use instead of the physical space [16, 17].

Although the use of a virtual library is beneficial in that patients can access the library anywhere there is internet, if patients must use virtual health libraries without the option of speaking to a librarian in person, then the virtual library only leads to a further increase in the digital divide. Even if patients have access to the internet, the digital divide is also impacted by connectivity, meaning that slow internet can have a negative impact on someone's use [18]. While the transition to virtual-only library services provided necessary safety measures for both library employees and library patrons while still meeting the information needs of patrons able to utilize the virtual services, a void in services was created for patrons dependent on the physical space for access to resources and guidance.

This inequity between virtual and physical availability of services prompted our investigation into the impact of COVID-19 closures and loss of physical space access on the number of health information requests at the Health Information Center (HIC) at the University of Tennessee. We expected the number of phone and email requests to increase since patients were not able to access the physical library. To determine the impact, librarians at HIC analyzed the patron use of the consumer health service before and during the COVID-19 pandemic to investigate the importance of access to a physical hospital library for patients and family members when it comes to providing health information.

## CASE PRESENTATION

The HIC is a patient and family centered library located inside the University of Tennessee Medical Center (UTMC), the region's only academic medical center, and is part of Preston Medical Library (PML), which is the library for the physicians, faculty, residents, nurses, and staff of the hospital. PML and the HIC have 5 faculty librarians and 5 full-time staff members. The HIC is located on the first floor of UTMC across from the pharmacy and provides patients and family members with 6 computers, comfortable seating, and over 400 health books, plus a leisure reading collection. Additionally, the HIC offers a free health information service through which patrons can request health information by calling, walking into the library, or emailing. Emailing includes a direct request using the library's email address or a web form located on the library's website. The information comprising a response can then be collected in person, emailed, or mailed, all of which are free.

The HIC experienced the same unknowns many other libraries faced during the COVID-19 pandemic. With the physical space closed, librarians focused on phone and email requests in order for patients to receive the same quality health information. Information packets were distributed through email or mail based on the user's preference.

## METHODS

We analyzed the data from our internal database of consumer health information requests from patients, family members, and the community, which has been maintained since the start of the consumer health service in the 1990s. The database includes name, address, zip code, and request details, such as interaction type, topic, and date. The request topic is then categorized using the National Library of Medicine's Medical Subject Heading (MeSH) terminology for consistency.

Data was exported from the internal database into an Excel spreadsheet and divided into three time periods: March 2018 to February 2019 (Phase 1), March 2019 to February 2020 (Phase 2), and March 2020 to February 2021 (Phase 3). Phases 1 and 2 were pre-COVID-19 while Phase 3 was during COVID-19 when the HIC was primarily open to the public through phone and email only. Researchers received IRB approval for this project (IRB #4740). Data was de-identified and duplicate entries were removed. The request topics were analyzed for each phase by using MeSH. The type of interaction, which included walk-in, phone, and email, was also analyzed for each phase. The three phases were compared to one another in the following ways to determine how Phase 3 affected the consumer health service: number of health information requests, number of requests by contact method, number of COVID-19-specific searches, and distribution of most requested topics.

To further analyze public interaction with the library, we reviewed questions asked by patients, family members, or the community to the library that were not a specific health information request, including questions about services, requests for any information other than health information, and general information such as printing and copying.

## RESULTS

**Figure 1:**
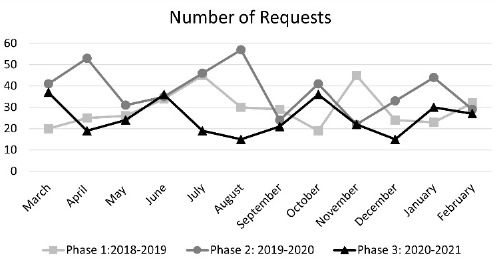
Number of Requests within 3 Phases

There were 887 health information requests in Phase 1, 1,011 requests in Phase 2, and 341 requests in Phase 3. Between Phase 1 and Phase 3, there was a 61.56% decrease in health information requests and a 66.27% decrease between Phase 2 and Phase 3. See Figure 1 for the number of requests within the three phases.

**Figure 2:**
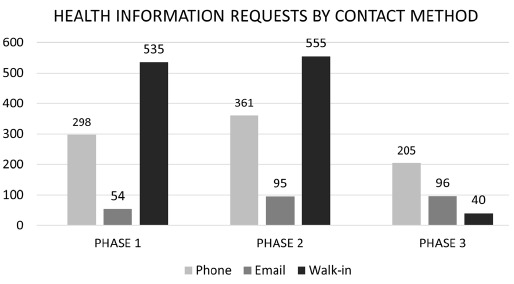
Health Information Requests by Contact Method

During Phase 1, there were 525 walk-ins of patients and/or family members to request health information. Phase 2 was relatively similar with 555 walk-in requests. During Phase 3, there were 40 walk-in requests, which came during the 7 weeks when the HIC reopened before closing again. The number of health information requests through email and phone varied: Phase 1 had 352 requests, Phase 2 had 456 requests, and Phase 3 had 301 requests. See Figure 2 for Phone/Email Requests vs Walk-Ins during the three phases.

Requested topics varied throughout each phase. The most common topics included diet, diabetes, heart, liver, and Alzheimer's. In Phase 3, there were 36 COVID-19 related health information requests. Diet was the most requested topic for Phases 1 and 2 while Coronavirus was the most requested topic in Phase 3, with diet being second.

Overall, all interactions with patients, family members, and the community, regardless of the type of information requested, drastically declined during Phase 3. Phase 1 had 5,128 interactions and Phase 2 had 4,207 interactions while Phase 3 only had 547 interactions.

During Phase 3 of the study, the library was closed to patients and family members, except for three weeks in June/July 2020 and four weeks in July 2021, which accounts for the small number of walk-ins during Phase 3.

## DISCUSSION

The closure of the physical space of the library to the public greatly impacted the number of health information requests and interactions with patients and family members. The total number of health information requests significantly decreased without the walk-ins, and the loss of these walk-ins were not made up by phone or email requests. From this study, the authors learned just how important the physical space is for providing patients and family members with reliable and accurate health information. We knew the loss of the physical space would have an impact on consumer health requests; however, the amount the requests decreased and that the email and phone requests did not increase surprised us. Phone and email requests not increasing during Phase 3 may be due to patrons of the service not having access to the internet or not knowing the library's phone number to request information. Patrons may not realize that the health information could be mailed for free to their homes and therefore did not make the request. Having no increase in phone or email requests may demonstrate how the majority of patrons were reliant on the physical space for their health information requests. Furthermore, the phone and email requests may have been mostly made by repeat patrons, which may explain why the numbers were slightly similar.

With some libraries facing budget cuts and potential closures, this study highlights the importance of physical library space for health care consumers in need of reliable and accurate health information. The decrease in requests with the closure of the physical space is an excellent example of what we know about information-seeking behavior from Harris and Dewdney's six principles [19]: “The (1) information needs arise from the help-seeker's situation; (2) the decision to seek help or not seek help is affected by many factors; (3) people tend to seek information that is most accessible; (4) people tend to first seek help or information from interpersonal sources, especially from people like themselves; (5) information seekers expect emotional support; and (6) people follow habitual patterns in seeking information” [20]. These principles have previously been applied to the health information needs, sources, and barriers of primary care patients [20] and here we apply them to patients and families in a hospital setting.

Patients come to the hospital or see their physician, and information given to them during their appointment may prompt the need for more information, reflecting the first principle [20]. Furthermore, family members may find themselves in a situation where they need health information on a family member's diagnosis or condition while they are in the hospital. Without the physical space, these family members may be unaware of a health library to find information and unable to fulfill their information need.

While the second principle acknowledges that information seekers seek help due to a variety of factors, the third principle says those who do choose to seek information want the most accessible option. The convenient location of the HIC in the same place that prompted the information makes it very accessible. With the physical space, patients are leaving their appointment and see the library, which is an immediate opportunity for them to request information. This ease of access may not be possible if hospitals solely focus on having virtual health libraries because the opportunity to get information at the point of need is lost. Without the physical space, patrons may instead look to the internet for their health information or to social media [21, 22] where they may find unreliable health information. This can then impact patrons in underserved and vulnerable populations who do not have equal access to information [5]. The physical location provides patrons with the opportunity to talk with a librarian and receive the health information they need in a timely manner without worrying about inconsistent internet or email and phone access. It also eliminates the barriers for those who cannot easily use technology. Virtual health libraries may not be reaching their most vulnerable patient population if there is no physical location.

The physical space of a library is particularly pertinent for the fourth and fifth principles since the staff at the HIC can provide information to the patients and caregivers in an emotionally supportive environment. A physical space provides the opportunity for patrons who prefer to do their own research to seek assistance from library staff if needed, whether that be help searching online or looking for a specific book to check out.

As previously mentioned, the majority of the phone calls and email requests during Phase 3 were made by repeat users, further highlighting the sixth principle that people follow the habitual patterns in information seeking. Phone and email requests did not increase, despite the physical space being inaccessible for much of Phase 3. It could be due to people retrieving the most accessible information (online or from other resources close to them) or not changing their information-seeking habits. In either case, more outreach could have helped to get more people using the phone or email service. Outreach to the hospitals' waiting rooms to inform patients and family members of the library and consumer health service was put on hold due to safety concerns [23], and we were not able to place new brochures and signage throughout the hospital during the pandemic. Another barrier to increasing phone call and email requests was that outreach to the rural communities and outside the hospital could not be completed during the pandemic. Without these outreach events, many patients were not made aware of the health information service. The physical space of a hospital library is essential to our community to ensure patrons are able to access and receive reliable health information in a way that matches their information-seeking behaviors.

Closing the physical HIC led to a significant drop in health information requests and interactions even though phone and email were still available. This study shows the vital importance of access to a physical location for hospital libraries in order to provide all patients with reliable and accurate health information that meets their information-seeking behavior needs. Furthermore, the physical library space is crucial to helping narrow the digital divide by offering in person services. The closure of hospital libraries due to budget cuts may be greatly impacting access to health information. The results of this research will lead to new marketing efforts for the HIC to increase patron awareness of the full scope of services as well as continuing to provide in-person information services. On a wider scale, hospital administrators and stakeholders should consider this research in budgetary planning and support for their libraries.

## LIMITATIONS

There are limitations to this study. There is not a dataset where the physical space was completely closed for the entire time period, as the library allowed walk-ins for three weeks in June/July 2020 and four weeks in July 2021. To protect anonymity, researchers could not analyze the data by patron name; therefore, we could not see how many or what kind of requests were being made by repeat users.

## Data Availability

Data associated with this article are available in the Open Science Framework at https://osf.io/u9yan/?view_only=e493802a24ee4156b5fb93968541b19f.
